# A Multi‐Ancestry TWAS in Alzheimer's Disease in African‐American, Hispanic/Latino and Non‐Hispanic White Populations

**DOI:** 10.1002/alz70855_106624

**Published:** 2025-12-24

**Authors:** Xinyu Sun, Makaela Mews, Nicholas R. Wheeler, Penelope Benchek, Christiane Reitz, Adam C. Naj, Jennifer Elizabeth Below, Brian W Kunkle, Anthony J Griswold, William S Bush

**Affiliations:** ^1^ Case Western Reserve University School of Medicine, Cleveland, OH, USA; ^2^ Department of Population and Quantitative Health Sciences, Case Western Reserve University, Cleveland, OH, USA; ^3^ Systems Biology & Bioinformatics, Department of Nutrition, School of Medicine, Case Western Reserve University, Cleveland, OH, USA; ^4^ Case Western Reserve Univeristy, Cleveland, OH, USA; ^5^ Gertrude H. Sergievsky Center, Taub Institute for Research on the Aging Brain, Departments of Neurology, Psychiatry, and Epidemiology, College of Physicians and Surgeons, Columbia University, New York, NY, USA; ^6^ Penn Neurodegeneration Genomics Center, University of Pennsylvania Perelman School of Medicine, Philadelphia, PA, USA; ^7^ Vanderbilt Genetics Institute and Division of Genetic Medicine, Vanderbilt University Medical Center, Nashville, TN, USA; ^8^ Dr. John T. Macdonald Foundation Department of Human Genetics, University of Miami Miller School of Medicine, Miami, FL, USA; ^9^ John P. Hussman Institute for Human Genomics, University of Miami Miller School of Medicine, Miami, FL, USA; ^10^ Department of Population & Quantitative Health Sciences, Case Western Reserve University School of Medicine, Cleveland, OH, USA; ^11^ Department of Population and Quantitative Health Sciences, Institute for Computational Biology, Case Western Reserve University, Cleveland, OH, USA

## Abstract

**Background:**

Transcriptome‐wide association studies (TWAS) extend Genome‐wide association studies by integrating gene expression data to explore the functional consequences of non‐coding variants. This study employs a multi‐ancestry TWAS approach to identify AD‐associated genes across groups, leveraging data from the Multi‐Ancestry Genomics, Epigenomics, and Transcriptomics of Alzheimer's (MAGENTA) Project. The study includes 224 African American ancestry (AFA), 235 European ancestry (EUR), and 298 Hispanic ancestry (HIS) individuals with both whole‐blood expression and genotype data.

**Method:**

We first analyzed the MAGENTA data to identify specific genetic variants that impact gene expression across the three groups using the Sum of Shared Single Effects (SuShie) method. We examined a region of 500 kilobases around each gene and SNPs with a minor allele frequency > 0.01. SuShiE uses a sparse modeling assumption, assuming no effects at most SNPs. This analysis produces credible sets which are highly likely to contain variants causally associated to gene expression. The study adjusted for covariates including age at exam, sex, AD status, principal components, cell‐type proportions, and PEER factors in the cis‐eQTL weight prediction model. We also applied MA‐FOCUS (Multi‐Ancestry Fine‐Mapping of Causal Gene Sets) to fine‐map TWAS associations at genomic risk regions with at least one significant gene.

**Result:**

Our analysis identified five AD‐related genes in three regions. Among those identified, the AD‐related *TREML2* was observed across all three groups. The SSW meta‐analysis of ancestry‐specific TWAS associations identified *TREML2* with a false discovery rate (FDR)<0.1. The gene's effect size directions were positive and consistent across all ancestries (SSW Z = 4.14; EUR Z=3.76; AFR Z=2.34; HISP Z=0.97), with a MA‐FOCUS PIP (posterior inclusion probability) of 0.88, suggesting robust association.

**Conclusion:**

This study highlights the importance of multi‐ancestry approaches in uncovering the genetic mechanisms influencing the molecular pathways involved in AD development. Zheng et al. found that the elevated TREML2 expression might exert pro‐inflammatory and proliferation promoting effects on microglia in an AD context. Our findings underscore the potential of *TREML2* as a target for further research and therapeutic intervention in AD. Increased sample sizes in studied populations will advance the identification of more AD‐related genes.

